# Juvenile hyperinsulinism in a Maine Coon kitten

**DOI:** 10.1177/20551169221136473

**Published:** 2022-11-23

**Authors:** Matthew Kornya, Anthony Abrams-Ogg, Dominique Comeau, Jeff Caswell

**Affiliations:** Ontario Veterinary College, University of Guelph, Guelph, ON, Canada

**Keywords:** Hyperinsulinism, hypoglycemia, insulinoma, nesidioblastosis, insulin

## Abstract

**Case summary:**

A 5.5 month-old intact male Maine Coon cat was presented to a referral hospital for a history of muscle fasciculations, lethargy and seizures associated with refractory hypoglycemia. Diagnostic testing for hypothyroidism, hyposomatotropism or hypoadrenocorticism, inborn errors of metabolism (ie, storage diseases and urea cycle disorders), infection or iatrogenic hypoglycemia were negative. An inappropriately high serum insulin level was noted in the face of marked hypoglycemia. The insulin:glucose ratio was 0.44 (<0.3) and the amended insulin:glucose ratio was 1268 (<30). Thoracic radiography and abdominal ultrasonography did not identify a cause for this elevated insulin level. Stabilization with a low, but adequate, blood glucose occurred with corticosteroid therapy, with further significant improvement with the addition of diazoxide. Peripheral neuropathy developed several months later, and concerns for quality of life led to humane euthanasia approximately 1 year after the initial diagnosis. Insulin levels remained high at the time of euthanasia. Necropsy found no gross lesions, though microscopic degeneration of the sciatic nerve and subjectively mildly increased size and number of pancreatic islets was noted. These findings were consistent with a diagnosis of congenital hyperinsulinism.

**Relevance and novel information:**

This is the first reported case of congenital hyperinsulinism in a cat and may parallel the diffuse form of hypoglycemic hyperinsulinism reported in humans and a single dog. It should be considered a differential diagnosis in kittens presenting for refractory hypoglycemia.

## Case description

A 5.5-month-old intact male Maine Coon cat was presented to a tertiary referral hospital for the evaluation of refractory hypoglycemia. The cat was noted to have had occasional paroxysmal full-body tremors for 1 week prior to presentation. These had progressed in the prior week to generalized tonic–clonic seizures, prompting presentation to the primary care veterinarian, who noted persistent hypoglycemia of 2.2–2.5 mmol/l (reference interval 4.4–7.7). An adrenocorticotropic hormone (ACTH) stimulation test and pre- and postprandial bile acids had been within normal limits (Table 1).

**Table 1:** Screening tests for causes of hypoglycemia

Test	Result	RI
Pre-ACTH cortisol (nmol/l)	<28	–
Post-ACTH cortisol (nmol/l)	250	>55
Preprandial bile acids (µmol/l)	0	0–3
Postprandial bile acids (µmol/l)	0	0–7
Fasting ammonia (µmol/l)	40	<60
IGF-1 (somatomedin C) (nmol/l)	146	12–92
Total T4 (nmol/l)	38	13–55
Free T4 (pmol/l)	29.5	18–52
cTSH (ng/ml)	0.1	NA

RI = reference interval; ACTH = adrenocorticotropic hormone; IGF-1 = insulin-like growth factor 1; T4 = thyroxine; cTSH = canine thyroid stimulating hormone; NA = not available

On presentation, the patient’s physical examination was unremarkable. Body weight was 3.3 kg, with a body condition score of 4/9. Venous blood gas analysis showed a blood glucose of 1.7 mmol/l (ABL Flex Radiometer). A serum biochemistry profile and a complete blood cell count were unremarkable other than hypoglycemia. Feline leukemia virus antigen and feline immunodeficiency virus antibody serology were negative by ELISA. Thoracic radiographs and an abdominal ultrasound examination were performed by a board-certified radiologist and resident, with no significant abnormalities noted.

To rule out hypothyroidism, portosystemic shunting, urea cycle disorders, hyposomatotropism or storage disease as causes of hypoglycemia, a thyroid panel, blood ammonia concentration and insulin-like growth factor 1 (Diagnostic Center for Population and Animal Health, Michigan State University) concentration were assayed (Table 1). These were considered unremarkable for a growing cat, with no evidence of a cause for hypoglycemia. A urine metabolic profile was performed to screen for inborn errors of metabolism (PennGen Metabolic Laboratory, University of Pennsylvania) such as storage diseases, enzymopathies or other metabolic disorders. This showed changes consistent with young age, with no other abnormalities (Table 2). A serum insulin concentration (Animal Health Laboratory, Ontario Veterinary College) was requested (collected at the same time as the initially documented hypoglycemia); however, the test has a turnaround time of several days.

**Table 2:** Results of a urine metabolic screen

Test	Result
Amino acids	Low, felinine present
Organic acids (hippurate/adipic acid)	Present
Carbohydrates	Negative
Glucose	Negative
Nitroprusside (cysteine)	Negative
Ketones	Negative
MPS spot test	Slightly positive (age related)
MMA spot test	Negative

MPS = mucopolysaccharide; MMA = methylmalonic acid

The cat was initially managed with intravenous (IV) fluids, antinausea therapy and antibiotics (ampicillin 22 mg/kg IV q8h) in case of a septic process. Blood cultures were not attained. No seizures were noted. IV dextrose was administered, with an initial supplementation of 5% at a maintenance fluid rate. A transient increase in blood glucose was noted up to 5.0 mmol/l, followed by a decrease to 3.5 mmol/l. Frequent small feedings were instituted, with a subsequent decrease in blood glucose to 2.9 mmol/l. The rate of fluid administration was increased from maintenance requirements to 1.5 × maintenance requirements, with no change in blood glucose noted.

Owing to refractory hypoglycemia and concerns for hyperinsulinism, dexamethasone (Dexamethasone 5; Vetoquinol) was administered IV at 0.1 mg/kg. This resulted in a marked increase in blood glucose to 7.4 mmol/l, allowing tapering of IV dextrose completely over the next 12 h. Antibiotics were discontinued once the results of imaging and bloodwork showed no evidence of infection.

Serum insulin levels were confirmed to be inappropriately elevated at 88 pmol/l, with a resultant increase in insulin:glucose ratio (IGR) and amended IGR (AIGR; Table 3). A diagnosis of hyperinsulinism of unknown cause was made. CT was recommended to examine for a potential insulinoma but was declined owing to cost concerns. As such, the cat was discharged with continued corticosteroid therapy (Prednisolone 5 mg tablets; Rafter Pharmaceuticals).

**Table 3:** Results of serial insulin and glucose evaluation

Value	Initial	Final	RI
Blood glucose (nmol/l)	1.7	2.1	
Serum insulin (pmol/l)	88	210	
IGR	0.44	0.80	<0.3
AIGR	1268	387	<30

RI = reference interval; IGR = insulin:glucose ratio; AIGR = amended IGR

Initially, the cat did well clinically, with a normalization of attitude and activity levels, and a resolution of fasciculation/seizure episodes. Prednisolone dose was tapered from 5 mg daily (~1.5 mg/kg) to 2.5 mg/day (~0.5 mg/kg). Growth of the kitten also reduced the dosage. Blood glucose measured at home with a handheld glucometer (Alpha-Trak) ranged from 2.5 to 3.7 mmol/l. Attempts to taper prednisolone to 1.25 mg led to a recurrence of muscle fasciculation within 48 h and a blood glucose reading of ‘LO’ (corresponding to <1.1 mmol/l), so the dose was increased to 2.5 mg q24h again.

Eight months later, the cat was noted to have developed an abnormal gait with a bilaterally partially plantigrade stance in the pelvic limbs and mild proprioceptive ataxia. Blood glucose levels measured at home in this time period ranged from 1.7 mmol/l to 3.1 mmol/l; however, other clinical signs of hypoglycemia were not appreciated. Differential diagnoses for this change included a hypoglycemic neuropathy, steroid-induced myopathy/laxity or unrelated peripheral neuropathies. Neurologic signs partially improved without alteration of the therapy.

Three months later, neurologic signs recurred, with progression to paresis and difficulty rising. A neurologic examination performed at an emergency hospital was suggestive of peripheral neuropathy affecting the hindlimbs, with a L4–S1 spinal lesion not excluded. Patellar and withdrawal reflexes were weak but present. Anal and tail tone were normal, as was the perineal reflex. An orthopedic examination was unremarkable. A serum biochemistry profile performed at this time was unremarkable other than a hypoglycemia of 2.2 mmol/L. Diazoxide therapy was initiated at a starting dose of 5 mg/kg orally q12h (compounded oral solution).

Diazoxide therapy resulted in a rapid and marked improvement in blood glucose, with a home glucometer reading of 7.3 mmol/l recorded 8 h after the initial dose. Despite this improvement, the owners noted a marked decrease in appetite, with no voluntary oral intake of food for 12 h after a dose was administered. Upon skipping a dose, appetite consistently improved. As this loss of appetite occurred at the lowest end of the dose range, continued diazoxide therapy was not considered feasible.

Alternative options, including somatostatin analogs or subtotal pancreatectomy, were discussed, but they were declined owing to cost and quality of life concerns, and humane euthanasia was elected. A blood sample was collected at the time of euthanasia, and a blood glucose (radiometer) and serum insulin level test was repeated. These confirmed persistent hyperinsulinemic hypoglycemia (Table 2).

A postmortem examination was performed by a board-certified anatomic pathologist and resident. Gross examination showed no significant lesions, with no evidence of a pancreatic mass or other neoplasm. The pancreas was collected in its entirety and sectioned for histopathology.

**Figure 1:**
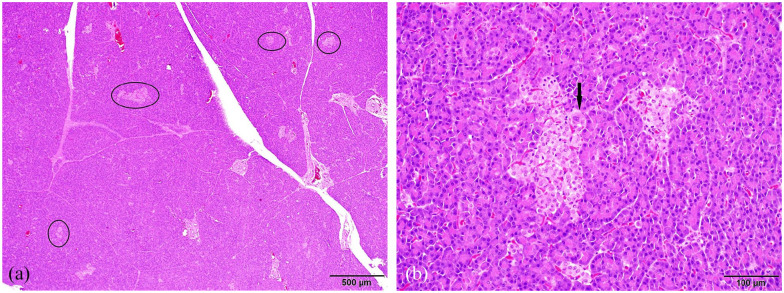
Representative sections of pancreas. (a) Throughout the pancreas, there was a subjective increase in the number and size of islets, with subtle variation in pancreatic islet size and shape (circled). (b) Rarely, the islets contained individual cells which had abundant deeply eosinophilic, finely granular cytoplasm and large, irregular nuclei up to 1.5 times the size of adjacent nuclei (arrow)

In all sections of the pancreas, there was a subjective increase in the number and size of islets, with subtle variation in islet size. Rarely, the islets contained individual cells which had abundant deeply eosinophilic, finely granular cytoplasm and large, irregular nuclei that were up to 1.5 times the size of adjacent nuclei (Figure 1). A small focus of ectopic pancreatic tissue was noted in the mesentery, comprised almost entirely of exocrine cells and some central hemorrhage.

**Figure 2:**
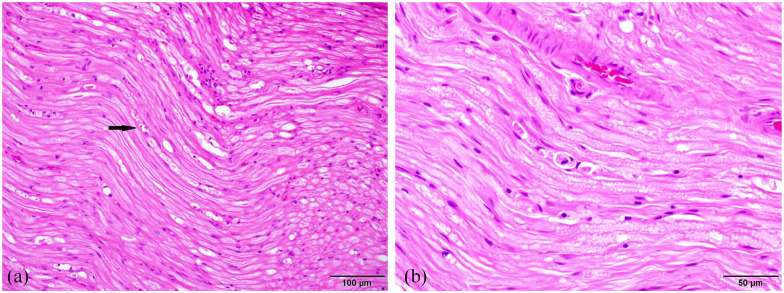
Representative sections of the sciatic nerve. (a) There were rare, large, clear vacuoles forming linear chains along the length of individual axons (dilated myelin sheaths – arrow) in the sciatic nerves. (b) The dilated myelin sheaths in the sciatic nerve occasionally contained macrophages (digestion chambers)

Within the sciatic nerve there were rare large, clear vacuoles that formed linear chains along the length of individual axons consistent with dilated myelin sheaths. Occasionally, these vacuoles contained macrophages, consistent with digestion chambers. There was an equivocal increase in the amount of immature fibrous tissue within the endoneurium (Figure 2).

No other clinically significant lesions were noted on microscopic examination of other organs. No neoplastic cells were present in any tissue, and the pituitary gland was microscopically unremarkable.

## Discussion

Hypoglycemia is commonly encountered in veterinary medicine and may lead to a variety of sequelae, including lethargy, seizures, transient or chronic neurologic deficits and, potentially, death.^1,2^ Common causes of hypoglycemia described in veterinary medicine include sepsis, liver failure, neonatal hypoglycemia, insulin overdose, metabolic disorders or neoplasia, though a multitude of genetic, endocrine and other causes have been described.^1,3,4^

In this cat, sepsis, liver dysfunction and metabolic disorders were excluded based on physical examination and diagnostic testing, though blood and urine cultures were not performed. The cat was considered to be too old and therapy-refractory for neonatal hypoglycemia, and the anamnesis and duration of clinical signs too long for iatrogenic accidental or malicious insulin overdose. Neoplasia was considered; however, non-insulinoma tumors producing hypoglycemia are generally associated with lower serum insulin levels.^5–7^ While insulinoma could not be fully excluded based on ultrasound examination alone,^8^ non-insulinoma neoplasia was further de-prioritized based on the normal ultrasound examination.^9^ The necropsy findings of possible islet hyperplasia with no neoplastic beta cells ruled out insulinoma and was considered strongly suggestive of congenital hyperinsulinism.^10–12^

Peripheral neuropathy has been described as a consequence of insulinoma and chronic hypoglycemia in humans, dogs and cats.^10,13^ It has been theorized that the neuropathy present in cases of insulinoma may result from metabolic derangements due to severe and prolonged hypoglycemia.^14,15^ The postmortem changes in the cat in our report are consistent with those previously described in hypoglycemic neuropathy.^16^

While the IGR and AIGR are traditionally thought of as tests for the presence of an insulinoma, in actuality they simply indicate an inappropriately high insulin level for a given blood glucose concentration.^11,17,18^ When measuring AIGR, if the blood glucose concentration is <30 mg/dl, the number 1 is used as the divisor to avoid negative values.^19^ Most authors have suggested that an AIGR >30 is diagnostic of an insulin-secreting tumor.^20^

In the cat in our report, both IGR and AIGR were significantly elevated, and the absolute insulin value was inappropriately high for a hypoglycemic animal. As such, regardless of the chosen method of interpretation, this cat demonstrated a hyperinsulinemic state.

Non-neoplastic causes of hyperinsulinemic hypoglycemia include nesidioblastosis, a non-neoplastic proliferation of beta cells,^21^ and congenital hyperinsulinism, a metabolic defect in beta cell function resulting in constitutive expression of insulin. There is significant overlap in the definitions of nesidioblastosis and hyperinsulinism. Nesidioblastosis was initially used to describe both juvenile and adult-onset hyperinsulinism; however, it is considered outdated in reference to the congenital form, where the term ‘hyperinsulinism’ is currently preferred and should be used to refer to adult-onset disease.^10,21,22^ The term ‘congenital hyperinsulinism’ may refer to both a state of increased non-neoplastic beta cell mass or constitutive production of insulin by non-neoplastic beta cells.^23,24^

Adult-onset nesidioblastosis has been described in a single cat.^21^ Juvenile-onset, congenital, non-neoplastic proliferation of beta cells has not been described in feline medicine. In humans, nesidioblastosis represents approximately 4% of cases of hyperinsulinemic hypoglycemia.^22^ It presents as a diffuse hypertrophy or hyperplasia of beta cells throughout the pancreas, and may represent a regenerative response secondary to previous pancreatic injury.^21^ The age of the cat and histologic features on post-mortem examination in this case were inconsistent with nesidioblastosis.

Congenital hyperinsulinism in humans is a rare disease but is the most common cause of persistent and severe hypoglycemia in childhood.^24^ In humans, this condition is often genetic and has been described in relation to mutations in the potassium ATP channel gene, or in genes regulating the metabolism of beta cells.^24,25^ This results in either dysfunctional potassium channels or alterations in the ATP:ADP ratio, both of which prevent potassium efflux. In 27–47% of humans, no genetic basis is found and the pathologic process is unknown.^12^

Both focal and diffuse forms of hyperinsulinism have been described, with the focal forms characterized by a clonal expansion of beta cells that most often express mutations of the potassium genes *ABCCC8*/*KCNJ1*; these are often clinically more severe and drug resistant.^25^ In the diffuse form, nuclear enlargement of beta cells is seen, often fourfold vs normal beta cells.^25^ In this case some nuclear enlargement was noted, though only to 1.5 times normal size. It is unclear if this represents the same process as described in humans, or simply normal variation.

In humans, a diagnosis of congenital hyperinsulinism entails demonstration of persistent hypoglycemic hyperinsulinism, exclusion of other causes, genetic testing and positron-emission tomography.^23^ In the only other reported veterinary case, congenital hyperinsulinism was diagnosed in a 3-year-old Shiba Inu on the basis of persistent hypoglycemia, exclusion of other causes, and no findings on contrast CT,^12^ similar to the diagnostic process in this case; however, histopathology was not performed as the dog responded well to therapy.

While many cases of human hyperinsulinism may be controlled with medical or surgical management, between 26% and 48% of patients are left with neurologic deficits, generally peripheral neuropathies, as a result of prolonged hypoglycemia even after control is attained.^24,25^ While the lesions in human hyperinsulinism are generally found in the central nervous system, a similar outcome was noted in this feline case in the peripheral nerves; however, electromyography was not performed to confirm this electrophysiologically.

## Conclusions

This report described a kitten with hypoglycemic hyperinsulinism, potentially as a result of congenital hyperinsulinism. Hyperinsulinism should be considered a rare differential diagnosis in kittens presenting with refractory hypoglycemia.
